# New Archaeozoological Data from the Fayum “Neolithic” with a Critical Assessment of the Evidence for Early Stock Keeping in Egypt

**DOI:** 10.1371/journal.pone.0108517

**Published:** 2014-10-13

**Authors:** Veerle Linseele, Wim Van Neer, Sofie Thys, Rebecca Phillipps, René Cappers, Willeke Wendrich, Simon Holdaway

**Affiliations:** 1 Laboratory of Biodiversity and Evolutionary Genomics, Katholieke Universiteit Leuven, Leuven, Belgium; 2 Royal Belgian Institute of Natural Sciences, Brussels, Belgium; 3 Anthropology Department, The University of Auckland, Auckland, New Zealand; 4 Groningen Institute of Archaeology (GIA), Rijksuniversiteit Groningen, Groningen, The Netherlands; 5 Cotsen Institute of Archaeology, University of California Los Angeles, Los Angeles, California, United States of America; University of Florence, Italy

## Abstract

Faunal evidence from the Fayum Neolithic is often cited in the framework of early stock keeping in Egypt. However, the data suffer from a number of problems. In the present paper, large faunal datasets from new excavations at Kom K and Kom W (4850–4250 BC) are presented. They clearly show that, despite the presence of domesticates, fish predominate in the animal bone assemblages. In this sense, there is continuity with the earlier Holocene occupation from the Fayum, starting ca. 7350 BC. Domesticated plants and animals appear first from approximately 5400 BC. The earliest possible evidence for domesticates in Egypt are the very controversial domesticated cattle from the 9^th^/8^th^ millennium BC in the Nabta Playa-Bir Kiseiba area. The earliest domesticates found elsewhere in Egypt date to the 6^th^ millennium BC. The numbers of bones are generally extremely low at this point in time and only caprines are present. From the 5^th^ millennium BC, the numbers of sites with domesticates dramatically increase, more species are also involved and they are usually represented by significant quantities of bones. The data from the Fayum reflect this two phase development, with very limited evidence for domesticates in the 6^th^ millennium BC and more abundant and clearer indications in the 5^th^ millennium BC. Any modelling of early food production in Egypt suffers from poor amounts of data, bias due to differential preservation and visibility of sites and archaeological remains, and a lack of direct dates for domesticates. In general, however, the evidence for early stock keeping and accompanying archaeological features shows large regional variation and seems to be mainly dependent on local environmental conditions. The large numbers of fish at Kom K and Kom W reflect the proximity of Lake Qarun.

## Introduction

The area within the borders of modern Egypt is important for the reconstruction of the spread of stock keeping over Africa. It served as a potential overland corridor through which (Near Eastern) domesticates passed before they reached other parts of the African continent [1]. However, it is also possible that coastal areas of northern Africa were part of the Mediterranean zone where the expansion of agricultural economies was accomplished through several waves of seafaring [2] as recent archaeological data suggest [3].

The geography of Egypt is largely determined by the river Nile. At present the Nile Valley is a narrow fertile zone breaking up the Eastern and Western Desert that stretch far beyond its banks. However, during the Early and Middle Holocene, most of northeastern Africa profited from a more humid climate compared to the present-day, with “green deserts” as a consequence [4]. Northeastern Africa is at the border of the Mediterranean rainfall zone and the Inter-Tropical Convergence Zone (ITCZ), with its summer monsoonal rains, which both have shifted through time and so have influenced the potential for human occupation [5].

The earliest communities on the African continent which used domesticated food resources are thought to appear at the latest during the 6^th^ millennium BC. They are mobile hunter-gatherer-livestock keepers from the Egyptian desert, mainly the Western Desert, who did not practice agriculture, but were using pottery and relying heavily on the exploitation of wild plants, and were thus very different from the earliest Near Eastern food producing societies [6], [7], [8], [9]. Because of these differences, the term Neolithic is sometimes avoided for the earliest African period of food production [10]. Nevertheless, we will be applying Neolithic here for the time period when food production has been attested. In this paper, food production refers to any type of exploitation of domesticated food resources, either plants or animals. All dates mentioned in the text are calibrated.

**Figure 1:**
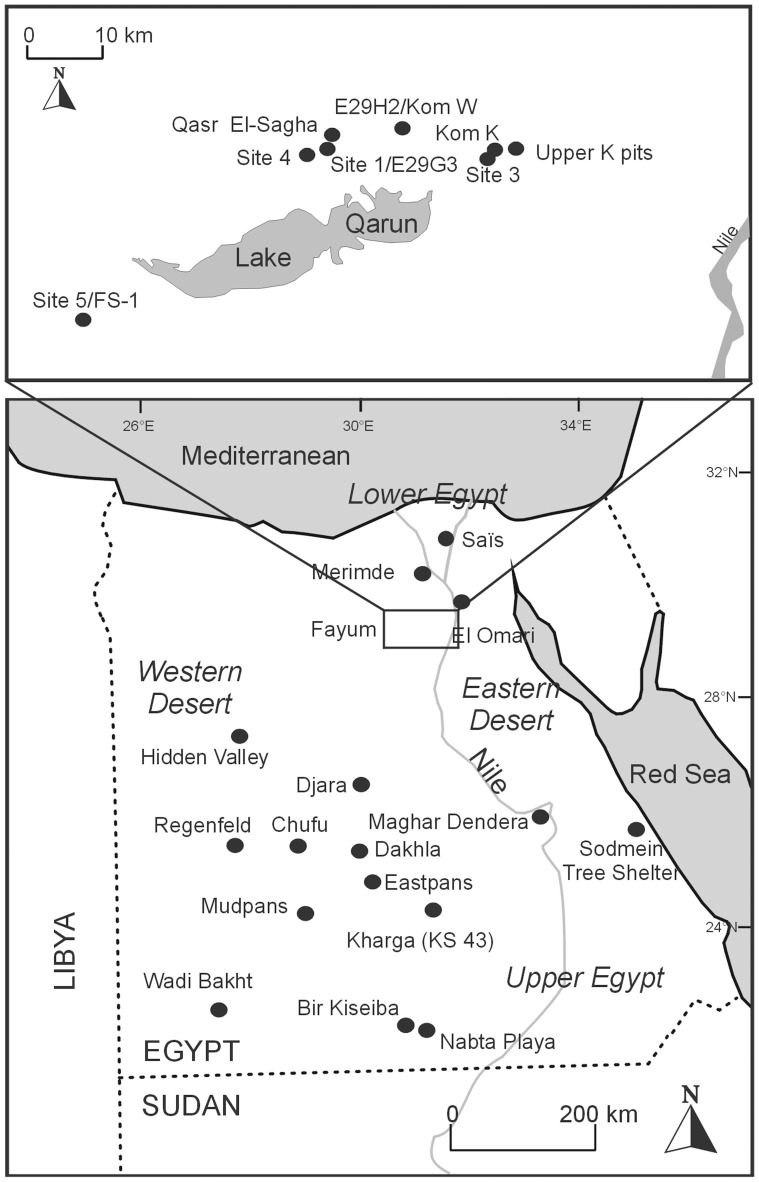
Map of Egypt indicating the localities mentioned in the text, with detailed map for the Fayum Oasis above.

In the first half of the 20^th^ century AD, the importance of the Fayum sites Kom K and Kom W was emphasised because of the early evidence of domesticated plants and animals they contained [11]. Together with Merimde Beni Salama [12] and Saïs [13] in the Nile Delta, the Fayum is the area with first evidence for both domesticated plants and animals in Egypt (Fig. 1). Several teams have worked on Neolithic sites in the Fayum and since 2006 investigations have been taken up again at Kom K and Kom W, by a team of the University of California, Los Angeles (UCLA, USA), the Rijksuniversiteit Groningen (RUG, The Netherlands) and The University of Auckland (New Zealand). These renewed excavations have yielded by far the largest faunal sample for the prehistoric period in the Fayum. Animal remains were studied before, but it will be shown that the large new samples represent a more firm, more detailed and less biased collection. Their study is therefore important to better understand the range of domestic species present, their relative (economic) importance, the nature of their exploitation and the seasons and duration of occupation of the sites.

In this paper, the archaeozoological data from the renewed investigations at Kom K and Kom W is first presented, against the backdrop of previous faunal studies on Fayum Neolithic sites. The dates at which domestic species appear and their relative importance compared to wild species in the Fayum is then compared to the available data from other northeast African sites in order to better understand the degree of regional variability that characterizes the northeast African Neolithic. The regional comparison begins by considering the 9^th^/8^th^ millennium BC Nabta Playa and Bir Kiseiba controversial evidence for locally domesticated cattle, and ends with the earliest Predynastic sites from the Nile Valley, dating to the second half of the 5^th^ millennium BC. Firstly, the physical evidence for domesticates is evaluated. Secondly, the location, age, quantity and circumstances under which different domestic species were first recorded are summarised. The focus is on food animals. Domestic cat (*Felis sylvestris* f. catus) and donkey (*Equus africanus* f. asinus) are therefore not discussed, even though the latter must have been economically important [14]. Although the domestic dog (*Canis lupus* f. familiaris) was presumably not consumed, data on this domestic species is mentioned, because of its possible role as a herding animal. An emphasis on the evidence for actual food production may obscure the diversity of Neolithic human societies. Therefore, the assessment aims to evaluate the variability in the concrete evidence for early food producing economies in Egypt, including variation in the parameters related to the mobility pattern of the human communities. This is relevant because the mobility pattern has been considered as one of the major differences between the earliest food production in Northeastern Africa (mobile) vs. the Near East (sedentary) [6].

### The stratified sites Kom K and Kom W in the Fayum

#### 1. Archaeological data and palaeoenvironment

Kom K and Kom W are both situated at the northern border of the Fayum Oasis, about 8 km north of Lake Qarun (Figure 1). However, in the Early and Middle Holocene the lake was significantly larger, and Kom K as well as Kom W must have been much closer to the shores during most of their Neolithic occupation. This occupation is dated between 4650 and 4350 BC, based on radiocarbon dates on charcoal from the sites [15]. Until the Aswan dam was built, Lake Qarun was connected to the Nile and it has been assumed that its water levels rose yearly in autumn, at the same time as the Nile levels, around the months August and September [16]. Throughout the Holocene, lake level fluctuations would have depended on Nile fluctuations, as well as on whether the connection remained open [16]. Contrary to the reconstructions of a fluctuating lake, Wenke et al. (1988) concluded from a plot by elevation of a selection of artifact types and faunal remains at the Neolithic site FS-1 that lake levels were stable. For the moment, there are insufficient geomorpohological data to be confident about the type of inundation regime of Lake Qarun in prehistory. The prehistoric occupation of the Fayum can be correlated with mid-Holocene increases in intensity of Mediterranean winter rainfall [5]. Winter rains probably resulted in more active wadi systems, and the retention of ground water in lower lying areas.

While most Fayum Neolithic sites are either shallow or surface sites, Kom W and Kom K have large stratified deposits. Kom W is the largest Fayum Neolithic site described up to now. Much of it was excavated in the 1920's [11]. New fieldwork at the site targeted the baulks left in situ during these early excavations, and previously unexcavated areas below the early excavations and at the foot of the kom (local synonym for tell). Kom K is situated about 10 km east of Kom W in the middle of modern farmland. The site is mostly famous because of the nearby Upper K pits, Neolithic granaries lined with basketry, approximately 800 m north of the kom, in which grains of emmer wheat (*Triticum turgidum* ssp. *dicoccon*) and hulled six-row barley (*Hordeum vulgare* ssp. *vulgare*) were recovered [11], [17]. Large numbers of ceramics and lithics have been recovered from both Kom K and Kom W. The sites have also yielded a large number of intact hearths and shallow depressions, but no postholes or substantial pits. It has been argued that the lack of house structures is due to the poor preservation of the perishable materials that were probably used [18], however there is no direct evidence that house structures ever existed. Inside some of the newly excavated hearths at Kom K, carbonized remains of cultivated plants have been found. From an analysis of lithics from both Kom K and Kom W, localized movements of the sites' occupants is suggested [19], [20].

#### 2. Previous archaeozoological studies in the Fayum

Faunal studies on Kom K and Kom W and other Fayum Neolithic sites were usually undertaken on rather small samples and/or surface material [11], [21], [22], [23], [24], [25] (Table 1). Surface material is biased towards hard, compact bones that resist weathering. Moreover, there is a higher risk of mixing with later material. Nevertheless, the earlier faunal studies have shown the presence of domesticated animals (cattle, sheep, goat, and probably also pig and dog) in the Fayum Neolithic, and the sites are therefore often cited in the context of early farming in Egypt and northeastern Africa in general [3], [26]. However, as clearly indicated by von den Driesch [22] and later emphasised by Brewer [24], [27], faunal samples from the Fayum Neolithic are predominantly composed of fish. The earliest researchers suggested that the Fayum Neolithic people were mainly dependent on fowling and fishing, rather than on agriculture, and therefore were representative of “an intermediary stage between hunting and agriculture” (M. Jackson cited in [11]). The renewed faunal study of Kom K and W provides the opportunity to expand the number of remains of domestic animals and to gather more details on the species composition and demographic profiles of the domestic livestock herds. Other informative aspects, including mainly size estimates of the fish are also reported.

**Table 1:** Summary of taxa identified during previous studies on fauna from the Fayum Neolithic.

Site	Kom W	Kom K	E29G3	E29H2 = Kom W	QS XI/81	QS IX/81	QS X/81	QS VI E/81	QS VII A/81	FS1-A	FS1-B	Site 1 = E29G3	Site 3	Site 4	Site 5 = FS1
Date (C14 uncalibrated BP) - for Kom K and Kom W see also Wendrich et al. 2010	-	-	5860+/−115	5810+/−115	6480+/−170	6380+/−60	6290+/−100	5650+/−70	4820+/−100	5160±70		see E29G3	-	-	see FS1
Reference	[11]	[11]	[21]	[21]	[22]	[22]	[22]	[22]	[22]	[23]	[23]	[24]	[24]	[24]	[24]
Surface (S) or excavated (E) material	E	E	S	S & E	E	E	E	E	E	S	S	S	S	S	S
**Fish**															
Mullets (Mugilidae)	-	-	-	-	-	-	-	-	1	-	-	-	-	-	-
Tiger fish (*Hydrocynus* sp.)	-	-	-	-	-	-	-	-	-	1	-	-	-	-	-
Barbel (*Barbus* sp.)	-	-	-	-	-	-	-	-	3	-	-	-	-	2	4
Catfish 1, clariid catfish (Clariidae)	-	-	-	-	12	50	5	-	97	13	-	154	277	415	2855
Catfish 2, Bagrid catfish (*Bagrus* sp.)	-	-	-	-	-	-	-	-	-	2	-	1	-	3	4
Catfish 3 (*Synodontis* sp.)	-	-	-	-	-	1	-	-	12	4	-	6	17	93	212
Catfish 4 (*Chrysichtys auratus*)	-	-	-	-	-	-	-	-	-	-	-	-	-	-	1
Nile perch (*Lates niloticus*)	-	-	-	-	-	5	-	-	13	8	-	26	13	73	96
tilapia (Tilapiini)	-	-	-	-	8	11	-	-	419	7	-	-	2	5	50
Pufferfish (*Tetraodon lineatus*)	-	-	-	-	-	-	-	-	1	22	-	1	-	-	274
**Identified fish**	-	-	**nd**	**nd**	**20**	**67**	**5**	**0**	**546**	**57**	**0**	**188**	**309**	**591**	**3496**
Unidentified fish	F	P	-	-	24	24	-	-	724	3257 g	9659 g*	-	-	-	-
**Reptile**	****	****	****	****	****	****	****	****	****	****	****	****	****	****	****
Snake (Serpentes) (intrusif?)	-	-	-	-	-	-	-	-	-	-	1.9 g	-	-	-	-
Lizard	-	-	-	-	-	-	-	-	-	-	0.5 g	-	-	-	-
Crocodile (*Crocodylus niloticus*)	P	-	-	-	-	1	-	-	-	-	3.5 g	-	-	-	-
Softshell turtle (*Trionyx triunguis*)	P	-	-	-	-	1	-	-	1	33 g	2385 g	334	132	32	336
**Identified reptile**	-	-	**0**	**0**	**0**	**2**	**0**	**0**	**1**	**0**	**0**	**334**	**132**	**32**	**336**
**Bird**	****	****	****	****	****	****	****	****	****	****	****	****	****	****	****
Whooper swan (*Cygnus cygnus*)	-	-	-	-	-	-	-	-	-	-	-	-	-	-	1
Duck (Anatidae)	-	-	-	-	-	-	-	-	-	-	-	16	1	-	7
Coot (*Fulica atra*)	-	-	-	-	-	-	-	-	1	-	-	-	-	-	-
**Identified bird**	-	-	**0**	**0**	**0**	**0**	**0**	**0**	**1**	**0**	**0**	**16**	**1**	**0**	**8**
Unidentified bird	-	-	-	-	-	1	-	-	-	32	-	-	-	-	-
Ostrich - eggshell	-	P	-	-	-	5	-	-	-	-	-	11	44	-	-
Other bird - eggshell	-	-	-	-	-	-	-	-	-	9	-	-	-	-	-
**Mammal**															****
**Wild**															****
Small rodent (intrusif?)	-	-	-	-	-	-	-	-	-	2	-	-	-	-	-
Hare (*Lepus capensis*)	-	-	-	-	-	-	-	-	-	5	4	-	-	-	-
Cat (*Felis* sp.)	-	-	-	-	-	-	-	-	-	-	1	-	-	-	-
Fox (*Vulpes* sp.)	-	-	-	-	-	-	-	-	-	2	2	1	-	-	-
Hippopotamus (*Hippotamus amphibius*)	4	-	1?	4	26	-	-	-	-	-	29	-	-	-	-
Dorcas gazelle (*Gazella dorcas*)	-	-	5	2	-	1	-	-	1	5	16	21	2	7	24
Hartebeest (*Alcelaphus buselaphus*)	-	-	-	-	-	-	-	-	-	-	2	9	3	1	5
Addax (*Addax nasomaculatus*)	-	-	-	-	-	-	-	-	-	1	-	-	-	-	-
Oryx (*Oryx dammah*)	-	-	-	-	-	-	-	-	-	-	2	-	-	-	-
Unidentified antelope	-	-	1	-	1	-	-	-	-	-	-	-	-	-	-
**Domestic**	-	-													
Domestic (?) pig (*Sus scrofa* (f. domestica?))	5	-	-	-	-	-	-	-	-	-	1	-	-	-	-
Sheep (*Ovis ammon f. aries*)	-	P	-	-	1	7	-	1	-	-	-	-	-	-	-
Goat (*Capra aegagrus* f. hircus)	-	-	-	-	-	1	-	-	-	-	-	-	-	-	-
Sheep or goat	8	-	142	16	4	38	-	-	3	22	125 g	149	3	5	39
Cattle (*Bos primigenius* f. taurus)	9	P	-	-	-	10	-	-	-	-	-	-	-	-	-
**Wild or domestic**	-	-													****
Wolf or dog (*Canis lupus* (f. familiaris))	5	-	1	2	-	1	-	-	-	3	8	1	-	-	19
Canidae	-	-	-	-	-	-	-	-	-	-	-	1	-	6	-
Small bovid	-	-	-	-	-	17	3	2	-	-	-	78	2	13	34
Wild or domestic cattle	-	-	25	-	2	-	-	-	-	2	1	51	2	-	35
Large bovid	-	-	-	-	2	2	-	-	-	-	-	-	-	-	-
Bovid	-	-	-	-	-	-	-	-	-	-	-	65	17	10	402
**Identified mammal**	-	-	**174**	**24**	**36**	**77**	**3**	**3**	**4**	**42**	**66**	**376**	**29**	**42**	**558**
Unidentified mammal	-	-	-	-	112	500	25	-	14	2221 g	12732 g	-	-	-	-

P: present, F: frequent, nd: no data, ?: identification uncertain; *No species identifications available.

Numbers are Numbers of Identified Specimens (NISP), where not available, weight is given in grams (g).

## Material and Methods

In total, the new excavations at Kom W yielded about 50,000 animal remains, and those at Kom K over 150,000 (Table 2). All fauna is from stratified deposits and was mainly collected through dry sieving on 2 mm meshes in the field. In addition, at Kom W, fauna recovered from the backfill of excavations in the 1920's was quickly scanned for the presence of domesticated species.

**Table 2:** Animal taxa identified from the renewed excavations at Kom K and Kom W.

INTRUSIVE	Kom K	Kom W
**Terrestrial snail**	3	-
**Freshwater shell**		
*Theodoxus niloticus*	103	47
*Bellamya unicolor*	14	-
*Lanistes carinatus*	2	-
*Valvata nilotica*	9	2
Bithyniidae	28	1
*Melanoides tuberculata*	28	23
*Cleopatra bulimoides*	607	17
*Lymnaea* cf. *natalensis*	4	-
*Planorbis planorbis*	19	-
*Gyraulus costulatus*	88	12
*Bulinus* sp.	36	-
*Corbicula consobrina*	138	-
Fossil shell	32	34
Fossil shark tooth	1	-
**Amphibian and reptile**		
Green toad (*Bufo viridis*)	2	-
Toad (*Bufo* sp.)	13	-
Frog or toad (Batrachia)	17	1
Small lizard	1	1
Snake (Serpentes)	4	-
**Bird**		
Small Passeriformes	2	-
**Mammal**		
Small rodent	14	2
ANTHROPOGENIC & UNIDENTIFIED		
**Shell**		
**Marine shell**		
*Nerita* sp.	1	-
Cowrey (*Cypraea* sp.)	3	-
Dove shell (*Columbella rustica*)	11	-
*Nassarius* sp.	3	-
Cone shell (*Conus* sp.)	4	-
Unidentified marine gastropod	1	4
**Freshwater bivalve**		
*Coelatura* sp.	9	-
*Spathopsis/Chambardia* sp.	50	2
*Mutela* sp.	20	-
**Identified shell**	**102**	**6**
Unidentified large bivalve	70	38
Unidentified bivalve	251	15
Unidentified gastropod	124	10
Unidentified mollusc	17	13
**Fish**		
Mullets (Mugilidae)	15	12
*Polypterus* sp.	13	-
*Hyperopisus bebe*	30	1
Elephant-snout fish (*Mormyrus* sp.)	4	4
Elephant-snout fishes (Mormyridae)	80	7
Tiger fish (*Hydrocynus* sp.)	11	8
Barbel 1 (*Barbus bynni*)	-	22
Barbel 2 (*Labeo niloticus*)	-	1
*Labeo* sp.	6	15
Barbel family (Cyprinidae)	351	758
*Alestes/Brycinus*	2	6
Catfish 1 (*Clarias gariepinus*)	1	-
*Clarias* sp.	29	1
Catfish 2 (*Heterobranchus* sp.)	1	-
Clariid catfish (Clariidae)	3667	851
Catfish 3 (*Auchenoglanis* sp.)	1	1
Catfish 4, Bagrid catfish (*Bagrus* sp.)	76	22
Catfish 5 (*Synodontis schall*)	24	9
*Synodontis* sp.	2072	279
Nile perch (*Lates niloticus)*	1812	698
tilapia (Tilapiini)	4042	2871
Pufferfish (*Tetraodon lineatus*)	20	7
**Identified fish**	**12257**	**5573**
Unidentified fish	29241	35237
**Reptile**		
Monitor lizard (*Varanus* sp.)	4	-
Softshell turtle (*Trionyx triunguis*)	27	7
**Identified reptile**	**31**	**7**
**Bird**		
Stork (Ciconiidae)	-	1
Duck (Anatidae size *A. crecca*)	-	1
Duck (Anatidae size *A. penelope*)	-	1
Duck (Anatidae)	15	2
Quail (*Coturnix coturnix*)	1	-
Water rail (*Rallus aquaticus*)	1	2
Coot (*Fulica atra*)	16	11
Plover (*Charadrius* sp.)	1	-
**Identified bird**	**34**	**18**
Unidentified bird	28	24
Ostrich (*Struthio camelus*) - eggshell	187	37
Other bird - eggshell	112	5
**Mammal**		
**Wild**		
Hare (*Lepus capensis*)	2	-
Cat (*Felis* sp.)	1	-
Fox (*Vulpes* sp.)	1	1
Small carnivore	4	-
Hippopotamus (*Hippotamus amphibius*)	4	-
Dorcas gazelle (*Gazella dorcas*)	4	-
Hartebeest (*Alcelaphus buselaphus*)	3	2
Wild bovid larger than gazelle	7	1
**Domestic**		
Pig (*Sus scrofa* f. domestica)	10	(2)*
Sheep (*Ovis ammon* f. aries)	14	5
Goat (*Capra aegagrus* f. hircus)	7	1
Sheep or goat	842	175
Cattle (*Bos primigenius* f. taurus)	14	-
**Wild or domestic**		
Wolf or dog (*Canis lupus* (f. familiaris))	64	7
Carnivore (size *Canis* sp.)	21	2
Small bovid (teeth)	2357	68
Small bovid (rest)	460	163
Large bovid	25	1
**Identified mammal**	**3840**	**426**
Unidentified mammal	107962	7496
**Total all**	**170520**	**54935**

*Two pig bones were found in the backfill of Caton-Thompson's excavations. No in situ remains.

Numbers are Numbers of Identified Specimens (NISP).

The animal bones from both Kom K and Kom W are usually small and fragmented. Fragmentation is especially high at Kom K, as reflected by the large number of small bovid tooth splinters (Table 2). The faunal remains sometimes have a grey-black colour that was initially interpreted as a consequence of burning, but is not always distinguishable from alterations to bone colours due to soil conditions. Less common are bones with a whitish colour mainly due to bleaching through exposure to the sun. Other bones have a more fresh appearance, suggesting that they have only briefly been exposed. At Kom W, material remaining in the baulks left by Caton-Thompson [11] was often embedded in an encrustation from a layer of evaporites that was removed by soaking in water.

The animal bone identifications were completed in the course of field work in 2006, 2007 and 2008, with the aid of bone atlases and a small reference collection built previously by M. Betti (Centre for Medieval Studies, University of Bergen, Norway) and T. Wake (Director of the Zooarchaeology lab, UCLA Cotsen Institute of Archaeology, Los Angeles, USA). In addition, one skeleton of each of the common most Nile fish species was brought to the field from the reference collection of recent skeletons in the Royal Belgian Institute of Natural Sciences (Brussels). The poor state of preservation and the high degree of fragmentation of the fauna from both sites seriously affected identification rates – a piece was considered as identifiable when the skeletal element was determined and attributable to a taxon below class level. For Kom K, 10.6% (16,215 specimens) of the bone remains were identifiable while for Kom W 12.4% (6,081 specimens) could be determined. In contexts with poor preservation, animals with larger bones will be represented by higher numbers of unidentified remains than species with small bones, because these large bones fall apart in many unidentifiable splinters. This explains the much higher proportion of unidentifiable mammal compared to fish remains in both Kom K and Kom W.

For quantification, numbers of identified specimens were counted (NISPs). Other quantification methods exist, but for all methods, including NISPs, the relationship with the living or dead animal population at the site is not straightforward [28]. NISPs were chosen because chances of interdependence, i.e. that several bones of one individual will be recovered and identified in an archaeological context, are small and because NISPs are the most simple to calculate, they are consistently available for comparative sites and have proven to give the best results for inter- and intrasite comparison [29]. Apart from skeletal element and taxon, indications of the sex and age of the animals were also recorded. When preservation allowed, fish standard length (SL i.e. the length from the tip of the snout to the beginning of the tail), was reconstructed by comparison with the bones of modern fish with known body length. Mammal and bird bones were measured according to the standard system developed by von den Driesch [30]. In addition, traces visible on any of the animal remains, for example of butchery or burning, were recorded and described.

All necessary permits were obtained for the described study, which complied with all relevant regulations. The permit number of the Egyptian Ministry of State Antiquities is 13/05/08 – 05. The bone remains of Kom K (KK) and Kom W (KW) are stored in the project's store room in the Fayum in Egypt, under the numbers FY06.1763-ee to FY07.8947-ee and FY06.821-ee to FY10.22594-ee, respectively. As part of a monograph that is currently in preparation, all data will be made available through the UCLA Digital Library, Los Angeles, as part of the project's final publications.

## Results

### Faunal data from Kom K and Kom W and other Fayum Neolithic sites

Part of the fauna from Kom K and Kom W, mainly the small shells but also various groups of small vertebrates, were probably not brought to the sites intentionally. They are listed separately in the species list as intrusive (Table 2). However, the large majority of the faunal remains recovered are likely anthropogenic and most probably represent food refuse. Only the anthropogenic fauna will be discussed in detail. The data are grouped together by site. Intrasite comparisons will be made in future publications, when more detailed archaeological reports are available.

In what follows, the major animal groups identified from Fayum Neolithic sites are first described in order of increasing abundance: marine shells, game animals, domestic animals and freshwater animals (see Table 3). The description is followed by a discussion of human mobility and details on the remains from the domesticated fauna that may be used as indications for the way these animals were being exploited.

**Table 3:** Relative proportions (%) of different animal groups at selected Fayum Neolithic sites.

Site	QS XI/81	QS IX/81	QS VII A/81	FS1-A	Site 1	Site 3	Site 4	Site 5 = FS1	Kom K	Kom W
Reference	[22]	[22]	[22]	[23]	[24]	[24]	[24]	[24]	This study	This study
Surface (S) or excavated (E) material	E	E	E	S	S	S	S	S	E	E
**Game animals**										
Crocodile	0	0.7	0.0	0	0.0	0.0	0.0	0.0	0.0	0.0
Monitor lizard	0	0.0	0.0	0	0.0	0.0	0.0	0.0	<0,1	0.0
Birds	0	0.7	0.2	0	1.9	0.2	0.0	0.2	0.2	0.3
Mammals (excl. carnivores)	48	0.9	0.2	12	4.7	2.7	3.3	4.7	0.2	<0,1
**Domestic animals (excl. carnivores)**	15	51.1	0.5	26	31.7	1.3	1.6	2.3	23,1/9,8*	6.9
**Freshwater animals**										
Fish	36	45.9	98.9	62	22.2	67.1	90.3	84.6	76.3	92.7
Softshell turtle	0	0.7	0.2	?	39.5	28.6	4.9	8.1	0.2	0.1
n	55	146	552	92	846	461	655	4130	16064	6015

? Only weights available and relative proportion therefore not calculated; *with/without small bovid teeth.

Remark: The table is based on numbers of identified remains in each category. Where the specimens could not be precisely identified, they were attributed to specific species using the same proportions in which these species occur in the precisely identified specimens. Taking for example, ‘small bovids’, which could be domestic or hunted, when a site yielded 2 bones identified as gazelle, 198 as sheep/goat, and 100 as small bovid, then 1 bone of the category ‘small bovid’ was counted with gazelle (hunted) and 99 bones with ovicaprines (domestic).

#### 1. Marine shells

The marine shells available for analysis with the other faunal remains from both K and Kom W (Table 2) point to contacts with the Red Sea, and perhaps also the Mediterranean. *Nerita* sp. found at Kom K and at Kom W – in the case of Kom W from the older material of Caton-Thompson and Gardner [11] – can only be found in the Red Sea, but all other marine shells recovered also occur in the Mediterranean [31], [32]. The other studies on Fayum Neolithic animal remains either do not mention shells [23], [24], [25] or describe freshwater bivalves only [21], [22].

#### 2. Game animals

Bird remains are not numerous in the Neolithic archaeological deposits in the Fayum (Tables 1–3), which is probably partially due to differential destruction of their relatively fragile bones. Nowhere do they represent more than 2% of the numbers of identified bones. Most of the taxa found at Kom K and Kom W are water birds, and include storks (Ciconiidae), ducks (Anatidae), water rail (*Rallus aquaticus*), coot (*Fulica atra*) and plover (*Charadrius* sp.). This fits the taxa identified from previously collected faunal assemblages. Coot is the most common species at both Kom K and Kom W.

Wild mammals are not very common at the Fayum Neolithic sites (Tables 1–3). Qasr El-Sagha XI/81 is the only site with a very high concentration of game, due to the presence of a large number of hippopotamus (*Hippopotamus amphibius*) bones, from one individual that was likely butchered and defleshed on the spot [22]. In the new material from Kom K and Kom W, wild mammals represent less than 1% of the identified vertebrate fauna. The identified taxa are cat (*Felis* sp.), fox (*Vulpes* sp.), hare (*Lepus capensis*), hippopotamus, dorcas gazelle (*Gazella dorcas*) and hartebeest (*Alcelaphus buselaphus*). All cattle bones fall in the size range of domesticated cattle and the presence of aurochs (*Bos primigenius*) (Table S1) is therefore not likely. In addition to these species, Redding (in [23]) mentions the presence of Barbary sheep (*Ammotragus lervia*), addax (*Addax nasomaculatus*) and oryx (*Oryx dammah*). All of the animals listed were probably found in or near the Fayum Oasis, judging from their modern distribution [33]. One of the smaller Fayum Neolithic sites investigated by Caton-Thompson and Gardner [11] is reported to have yielded elephant (*Loxodonta africana*) remains. This elephant may have been part of the relic population that existed in the Western Desert and that finally disappeared with increasing aridification [34]. One bone of crocodile (*Crocodylus niloticus*) was found. Monitor lizard (*Varanus niloticus*), another reptile, was also recorded, mainly at Kom K, where four bones were recognised. The frequency and species spectrum of wild game recorded for the Fayum Neolithic is very similar to that of Predynastic sites in the Nile Valley of Upper Egypt, where it is interpreted as evidence for opportunistic hunting close to the habitation areas [35].

#### 3. Domestic animals

The oldest evidence for domestic animals in the Fayum from Qasr El-Sagha XI/81, goes back to 5400 BC. At the site, one sheep bone and four bones that could not be specifically attributed to either sheep or goat were recorded. Slightly younger in age is IX/8, dated to ca. 5200 BC, where sheep, goat as well as cattle have been identified. The contemporary site X/81 yielded only a very small faunal sample from which no bones could be reliably attributed to domesticates.

After fish, remains of domestic animals are the second most numerous at the Fayum Neolithic sites, although there is considerable variation in their relative proportions (Table 3). Taphonomic factors probably explain many of the differences that can be observed. For instance, the high number of remains of livestock at Kom K compared to Kom W is a consequence of the large number of tooth fragments at the former, a result of higher fragmentation due to poorer preservation. However, even when the tooth fragments are excluded, domesticates are more common at Kom K than at Kom W (Table 3).

In all faunal samples, caprines are the most numerous domestic animals (Table 4). Very few caprine bones from Kom K and Kom W could be identified to species level, but both sheep (*Ovis ammon* f. aries) and goat (*Capra aegagrus* f. hircus) were recorded at the two sites. Sheep is predominant and this fits with previous observations (Table 4). Remains of domestic cattle (*Bos primigenius* f. taurus) are not common at Fayum Neolithic sites. This is the case at Kom K where cattle are very rare relative to the size of the faunal sample from the site. At Kom W the species is completely absent (Table 4). The domesticated status of caprines and cattle is confirmed by the measurements taken on remains from these animals (Table S1).

**Table 4:** Remains of domesticated animals at Fayum Neolithic sites.

Site	E29G3	E29H2 = Kom W	QS XI/81	QS IX/81	QS VI E/81	QS VII A/81	FS1-A	FS1-B	Site 1	Site 3	Site 4	Site 5 = FS1	Kom K	Kom W
Reference	[21]	[21]	[22]	[22]	[22]	[22]	[23]	[23]	[24]	[24]	[24]	[24]	This study	This study
Surface (S) or excavated (E) material	S	S & E	E	E	E	E	S	S	S	S	S	S	E	E
Sheep/goat	142	16	5	46	1	3	22	?	149	3	5	39	842	175
of which sheep	-	-	1	7	1	-	-	-	-	-	-	-	14	5
of which goat	-	-	-	1	-	-	-	-	-	-	-	-	7	1
Cattle	25	-	-	10	-	-	2	1	51	2	-	35	14	-
Pig	-	-	-	-	-	-	-	1	-	-	-	-	10	-(1)

?only weights available; (1): in situ remains only - two pig bones were found in the supposed backfill of Caton-Thompson's excavations.

Numbers are Numbers of identified specimens (NISP).Only precisely identified remains of sheep, goat, sheep/goat and cattle have been retained here.

**Figure 2:**
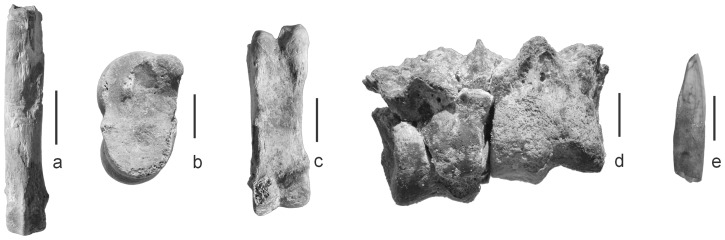
Selection of bones from Kom K and Kom W of each of the major domesticated animals. (a) Dog or wolf metapodial with cut marks (KK05-44) - volar view, (b) Sheep talus (KK03-27) - medial view, (c) Goat phalanx 1 (KK06-09) - volar view (d) Cattle distal metapodial (KK03-06) - dorsal or plantar view (e) Pig lower incisor (Kom W-back fill) - lateral view. Scale bar  = 1 cm. Short legend: Bones from Kom K and Kom W of each of the major domesticated animals.

Remains of pig (*Sus scrofa* (f. domestica)) were identified in the new material from both Kom K and Kom W. For Kom W, pig remains were absent in the undisturbed, stratified deposits, but one piece of pig skull and one lower incisor (Fig. 2e) were recorded during inspection of the back fill of previous excavations. Caton-Thompson and Gardner [11] report pigs from Kom W and Wenke et al. [23] mention a pig tooth from their excavations. Redding (unpublished data) found two pig maxillae and a distal tibia among diagnostic bones collected from the surface of the surroundings of the site FS-1. None of the Fayum Neolithic pig remains could be measured and it is therefore impossible to ascertain their domestic status on an osteological basis [36]. However an indirect argument for domesticates is provided by the absence of suids in Epipalaeolithic faunal assemblages of the Fayum [21], [23], [24], [25] (and Veerle Linseele and Wim Van Neer, unpublished data). There is no bone evidence for wild boar in Egypt before the Neolithic and it seems most likely that later finds and historically documented wild boar populations represent feral domestic pigs [37].

Various canid bones were also present in the new assemblages from Kom K and Kom W (Table 2). It could not be ascertained whether they derive from domestic dog (*Canis lupus* f. familiaris) or wolf (previously described as golden jackal (*Canis aureus*) but then reclassified cf. [21], [38], [39]). DNA analyses would be necessary to bring certainty about species identification [40]. However, identification as dog is considered more likely in view of the general predominance of domestic over wild mammals at the sites. One of the other sites in the Fayum have canid remains that could be identified to species level (Table 1), although usually domestic dog was considered the most likely candidate. Skins of canids appear to have been removed, inferred from a canid metapodial from Kom K with cut marks on its distal end and on the diaphysis (Figure 2a).

#### 4. Freshwater animals

Although the domestic animals found at Fayum Neolithic sites usually get most attention, fish are actually the most common animal group, as previously shown by von den Driesch [22] and Brewer [24], [25]. They represent up to 99% of the numbers of identified faunal remains (Table 3). In addition to fish, an aquatic reptile, the softshell turtle (*Trionyx triunguis*), is also present. Its numbers are particularly high in the surface sites (Table 3), which is not surprising as its carapax and plastron are very sturdy bones that preserve well and are easy to recognise.

The larger faunal dataset from Kom K and Kom W produced more fish species than from other Neolithic sites (Table 1 and 2). The species distribution is also much less biased than the surface sites, where large bones predominate. Different types of aquatic habitats are indicated [41]. Most fish are from shallow waters, especially represented by clariid catfish (Clariidae) and tilapia (Tilapiini) but including also fish from the Barbel family (Cyprinidae). Other species are typical of well-oxygenated water, tiger fish (*Hydrocynus* sp.), bagrid catfish (*Bagrus* sp.), *Synodontis* catfish and Nile perch (*Lates niloticus*). Few remains of fish of marshy, vegetated aquatic environments have been found, with these represented only by the genus *Polypterus*, indicating that such environments were probably not very common. Today Lake Qarun is saline and contains many marine fish species that have been introduced for commercial purposes [42], [43]. The rich spectrum of fresh water species can only have existed thanks to a (periodic) connection to the Nile, during and/or before the Neolithic.

In addition to fish and turtles, large freshwater bivalves were present with *Coelatura* sp., *Spathopsis/Chambardia* sp. and *Mutela* sp. identified. In addition, von den Driesch [22] found Nile oyster (*Etheria elliptica*) at Qasr El-Sagha VI E/81. The shells may have been collected for consumption while some worked specimens from the Kom sites indicate that they served as raw material.

#### 5. Seasonality and degree of mobility

The bird fauna from Kom K and Kom W is small but relatively rich in taxa, among which coot is the most common. Egypt today has rare resident populations of this bird, however, the country gets many coots as winter visitors, meaning that the species is most abundant from mid-September to early April [44]. Other bird taxa, especially the ducks, are probably winter visitors to the area too, although most have local breeding populations as well [44]. Like the present Lake Qarun, the Neolithic lake in the Fayum may have been one of Egypt's most important areas for migratory and water birds [43].

In arid areas, wild game usually congregates in (seasonal) periods of droughts in places where pasture and drinking water are still available. In such periods they are easier to hunt. If we can infer Mediterranean winter rainfall during the prehistoric occupation of the Fayum [5], then local rainfall would have been most restricted in the summer months. However, near the lake shores vegetation and water would still have been available, and the summer is also the period of the Nile floods. Winter rains may have caused lower lying areas to have a greater retention of ground water. It seems likely therefore that in the Fayum itself resources remained sufficiently available throughout the year and this may have attracted game animals from the surrounding areas in the dry summer and autumn months.

Information on ages at death of the domesticated animals is not precise enough to connect it to seasonal peaks in slaughtering (see below). In contrast to cattle and sheep/goat, pigs are typically associated with settled farmers [45]. Hence, in view of the poor numbers of pig bones from previous studies in the Fayum, we found it particularly important to confirm the presence of this domesticated species. Pigs are not suited for pastoral economies in arid environments given their high water requirements and their inability to feed on cellulose-rich plants, which means that they are typically kept close to the settlements [46]. Pig also does not provide its owners with milk or other secondary products, which are very important to current day pastoral communities [9].

Tilapia and clariid catfish, the two most common fish taxa, are mainly represented at Kom K and Kom W by large, sexually mature specimens, with average standard lengths of about 30 cm and 60 cm respectively Table S2). These must have been captured when they were spawning in shallow waters [41]. If the lake was seasonally inundated, fishing was probably mainly carried out during the yearly rise of the lake levels, and the months following, presumably August-September or slightly later. Clariids are present for only a few days in the shallow margins of flooded areas and will disperse afterwards [47]. Tilapia, however, are repetitive breeders [48]. Very conspicuous circular nests are made by all tilapia species living in Egypt that allow them to be easily located [48]. Despite systematic sieving on 2 mm meshes, small individuals of clariids and tilapia are almost completely absent from the recovered assemblages. These small fish can typically be harvested from residual pools that are formed when flood waters recede [41]. Either these fish were not exploited or no such pools existed along the lake. If Lake Qarun did not flood seasonally, then high solar radiation and sunshine hours in summer may have triggered spawning [49], [50]. In any case, it is clear that the spawning fish must have been a predictable food resource, as spawning always happens in certain parts of the year only. The fact that the animals reproduce in shallow, inshore waters means they could be easily harvested, even by hand [41]. Data on fish sizes are missing for the other Fayum areas however Brewer [24], [25] analysed growth rings on the pectoral spines of clariid catfish and correlated growth phases with periods of high temperatures. Although we believe that nutrient availability of flooded areas may have been the main variable determining the growth rates, his conclusion that fishing was mainly practised in May-June (temperatures similar to those of modern Egypt's late spring or early summer), is not incompatible with our hypothesis that fishing happened mainly in the late summer months based on the predominance of adult/spawning fish.

From the discussion above (see also the summary in Table 5), it appears that the exploitation of certain animal resources, especially fish, had seasonal peaks. However, this does not mean human groups were absent during other seasons. In fact, restricted mobility – people moving within the Fayum area – is suggested based on the study of lithics from Kom K and Kom W [19], [20], a result compatible with the presence of pigs.

**Table 5:** Summary of indications from palaeoenvironmental and faunal data for seasonality and mobility during the Fayum Neolithic.

Environment	Local rainfalll	Highest in winter
	Lake levels	Inundations in summer? (August-September)
	Temperature	Highest in summer
	Solar radiation	Highest in summer
Animal resources	Birds	Mostly in winter (mid-September to early April)
	Game	Possible peak in summer?
	Pig	Indicator for low mobility
	Fish	Peak in fishing during spawning season (Triggered by inundations? Or by rising temperatures and solar radiation?)

#### 6. Stock keeping in the Fayum: its earliest appearance, nature and (economic) importance

The new faunal studies at Kom K and Kom W have significantly increased the numbers of identified bones of domesticated animals for the Fayum Neolithic (Table 4). The earliest remains date to ca. 5400 BC but most evidence, including that from Kom K and Kom W, is from the mid-5^th^ millennium BC. Domesticated animals are present at nearly all prehistoric sites in the Fayum dating after 5400 BC, but numerically fish are predominant. This is also true of the preceding Epipalaeolithic period [21], [23], [24], [25] (and Veerle Linseele and Wim Van Neer, unpublished data). In fact, as has been pointed out by Brewer [24], [25] and Wetterstrom [26], the species spectrum of the fauna of all prehistoric sites in the Fayum is very similar apart from the presence of domesticated animals at the later sites.

Among the domesticated animals, remains of sheep/goat are most common. Although scant, the available evidence points to higher numbers of sheep than goat. For the total of the previously studied material, the proportion is 9∶1, for the new material from Kom K and Kom W the proportion is 2∶1 and 5∶1 respectively (Table 4). A higher proportion of sheep than goat in African livestock herds is usually taken as an indication of good grazing areas, since sheep are grazers that need relatively good pasture, while goats are browsers that can live of a more varied diet [51]. Cattle need more drinking water and better pasture than caprines [51] and their frequencies among present-day stock-keepers are therefore proportional to the availability of these resources. The lake must have provided sufficient drinking water year round, but the low numbers of cattle may indicate the absence of suitable grazing areas for these larger herbivores.

No data on ages at death of domesticated animals are available for the other Fayum Neolithic sites. One fetal or neonate caprine bone, a scapula, was found at Kom K. At Kom K 12 and at Kom W three caprine bones were classified as juvenile, based on the fact that they are not well ossified (see the summary of ageing data in Table S3). A precise age at death cannot be determined from this, but they must have belonged to animals less than one year of age. Slightly more than half of all caprine phalanges found are unfused (Kom K: 26 fused vs. 27 non fused; Kom W: 13 unfused vs. 6 fused), which corresponds to animals with an absolute age under 13–16 months [52], but not much younger as the bones are well ossified. Caprines kept for meat are mainly slaughtered between the age of 1 to 3 years, around the time they reach their maximal size, as this gives the maximum output for the least input [53]. The ages observed at Kom K and Kom W may be the result of a high natural mortality, combined with slaughtering mainly for meat. Because of the paucity of pig remains and the fragmentary state of these remains, data on ages at death are not available for pig. Hardly any data on ages at death are available for the cattle either. Kom K yielded one unfused distal metapodial, two fused first and one fused second phalanx. No reliable inferences can be made on this basis about slaughtering strategies, a recurrent problem for African archaeological sites that prevents demonstrating the use of cattle for secondary products like milk. Despite the lack of direct evidence, it has long been suspected that cattle milk was exploited by stock keeping communities of Africa from very early times [54], [55], [56], [57]. In addition, from the Libyan Sahara there is now evidence through residue analysis of pottery for extensive processing of dairy products, although unspecified whether from cattle or caprines, during the Middle Pastoral period (approximately 5200–3800 BC) [58]. As is the case with the Fayum, the number of bones from domesticates at the site is extremely low compared to fish (Francesca Alhaique, Wim Van Neer, Monica Gala and Savino di Lernia, unpublished data).

### Evidence for early stock keeping in other parts of Egypt

As indicated above, the predominance of fish in the faunal remains from Kom K and W likely reflects the proximity of Lake Qarun. This needs to be considered when assessing the relative economic importance of domestic stock in the Fayum but also when assessing the chronology and relative abundance of domestic animals within the wider northeast African region. The introduction of domestic animals need not have occurred in the same way or at the same time in all places within the region, so in understanding the relative significance of the Fayum evidence it is important to document how similar or different the material from Kom K and Kom W is to other sizeable faunal assemblages. In this section we consider other early food productions sites in Egypt, organised by geographic region: the Western Desert, the Eastern Desert and the Nile Valley, further subdivided into the Nile Valley of Lower and of Upper Egypt. Absolute dates and the natural environment of the sites are briefly described. References to the dates of sites mentioned will only be included when missing from the summary of radiocarbon dates for most Egyptian Neolithic sites in Phillipps et al. [5]. To the degree that the data allow, we summarize results from previous studies that allow direct comparisons with the Fayum data reported here. Summaries are provided of the domestic animal species present and their relative importance in the faunal assemblages, on the relative contribution of economic products from agriculture, as well as seasonality and mobility. Table 6 contains an overview of the faunal data for the sites mentioned.

**Table 6:** Relative proportions (%) of different animal groups of Egyptian Neolithic and (early) Predynastic sites and numbers of domesticated animals identified.

		Western Desert																Eastern Desert		Lower Egypt								Upper Egypt
	Site	Djara	Hidden Valley site	KS 43	Nabta/Bir Kiseiba EN	Nabta/Bir Kiseiba MN	Nabta/Bir Kiseiba LN	Regenfeld I	Chufu I	Mudpans I	Glass area I-II	Regenfeld II	Chufu II	Mudpans II	Chufu III	Westpans III	Wadi Bakht III	Sodmein	Tree Shelter	Merimde I	Merimde II	Merimde III	Merimde IV	Merimde V	Saïs M-LN	Saïs LN	El-Omari	Maghar Dendera 2
	**Reference**	[59]	[61]	[65]	[69]	[69]	[69]	[72]	[72]	[72]	[72]	[72]	[72]	[72]	[72]	[72]	[73]	[76]	[80]	[12]	[12]	[12]	[12]	[12]	[85], [86]	[85], [86]	[89]	[92]
	**Chronological phase**	2	2	3	1	2	3	1	1	1	1-2	1-2	1-2	1-2	3	3	1-3	2	2	3	3	3	3	3	3	3	3	3
%	**Fish**	0.0	0	0.2	0.0	0.0	0.0	nd	nd	nd	nd	nd	nd	nd	nd	nd	nd	0.0	12.9	12.7	42.8	25.5	41.2	46.0	96.7	20.2	66.3	85.9
	**Domestic animals (excl. carnivores)**	0.9	22	94.0	2.2	19.4	38.0	0	0.0	0	0	0	0	0.0	0	0	33	66.5	47	85.2	55.3	70.9	55.4	49.9	2.1	54.9	28.5	11.9
	**Hunted animals**																											
	Reptiles	0.0	0	0.0	0.0	0.3	0.0	nd	nd	nd	nd	nd	nd	nd	nd	nd	nd	0.0	0.0	0.4	0.1	0.6	0.2	0.3	0.0	0.0	0.8	0.5
	Birds	0.2	3	0.0	0.0	0.0	0.0	nd	nd	nd	nd	nd	nd	nd	nd	nd	nd	0.0	0.0	0.8	1.0	1.2	1.0	1.4	0.1	0.4	0.5	0.6
	Mammals (excl. carnivores)	98.9	75	5.8	97.8	80.3	62.0	100	100.0	100	100	100	100	100.0	100	100	67	33.5	40	0.9	0.8	1.8	2.2	2.4	0.0	0.3	3.9	1.2
	n	470	68	2984	3102	797	698	68	104	14	96	12	27	2519	11	26	15	167	70	1556	6765	165	11010	2699	5056	769	1532	1056
	**Domestic animals (excl. carnivores)**	0.9	22.1	94.1	2.2	19.4	38.0	0	0.0	0	0.0	0.0	0.0	0.0	0.0	0.0	33.3	66.5	54.1	97.6	96.6	95.1	94.3	92.5	96.3	98.8	84.7	84.1
	**Hunted animals (excl. carnivores)**	99.1	77.9	5.9	97.8	80.6	62.0	100	100.0	100	100	100	100	100.0	100	100	67	33.5	46	2.4	3.4	4.9	5.7	7.5	3.7	1.2	15.3	15.9
	n	470	68	2979	3102	797	698	68	104	14	96	12	27	2519	11	26	15	167	61	1359	3868	123	6469	1457	108	427	516	149
NISP	**Domestic animals**																											
	Cattle	-	-	587	56	35	83	-	-	-	-		-	-	-	-	5	-	-	250	759	30	1348	335	48	159	152	11
	Sheep/goat	2	15	1769	-	120(1)	182(1)	-	-	-	(2)	-	-	-	-	-	(3)	10	7	835	1627	25	1919	366	9	28	102	27
	of which sheep	1(4)	min. 1	46	-	-	-	-	-	-	-	-		-	-	-	-	-	-	138	208	34	5	239	4	1	-	4
	of which goat	-	min. 4	19	-	-	-	-	-	-	-	-	-	-	-	-	-	1	2	2	7	1	-	23	-	1	-	3
	Pig	-	-	-	-	-	-	-	-	-	-	-	-		-	-	-	-	-	241	1352	62	2835	646	47	235	183	-
	Dog/wolf	-	-	5/-	-/26	4/29	17/13	-	-	-	-	-	-	-/18	-	-	-	-	-	20/-	162/-	7/-	171/-	38/-	1	4	6/-	-

Phases: 1: older than the 6th mill. BC; 2: ca. 6th mill. BC; 3: ca. 5th mill. BC

(1): mainly sheep, including probably Barbary sheep; (2) but 3 sheep bones have been found in an other collection at this site than the one included here; (3) but 1 bone probably from later period; (4): identification uncertain

Based on Numbers of Identified Specimens (NISP). Only precisely identified remains of sheep, goat, sheep/goat and cattle are retained here. For similar data on younger Predynastic sites see Linseele et al. [92]

Remark: As in Table 3, the proportions are based on number of identified remains in each category. Animals considered as intrusive have not been included. Where the specimens could not be precisely identified, they were attributed to specific species using the same proportions in which these species occur in the precisely identified specimens.

#### 1. Western Desert

A concentration of prehistoric sites occurs in the Djara depression, on the Egyptian limestone plateau [59]. The paleo-ecological evidence from the depression points to a more humid climate in the Early and Middle Holocene with the influence of both winter rains from the north and west and the summer monsoonal rains from the south. The prehistoric occupation includes an Epipalaeolithic phase (7700–6700 BC) preceding the Djara A (6500–5900 BC) and Djara B (5800–4500 BC) phases Fish remains have not been found. The fauna mainly consists of desert antelopes (addax, oryx and gazelle) and many bovids that could not be identified more precisely [60]. The identified faunal remains (ca. 470) include a left lower second molar of a sheep or goat, directly dated to ca. 4900 BC, and an ulna from Djara A, that while originally identified as sheep [59], is not diagnostic and the identification must remain tentative [60]. These two bones are the only reported remains of domesticated animals at Djara. For Djara B, the large numbers of grinding implements recorded point to the importance of wild plants in the human diet at this time, but no cultivated plants have been identified [59]. A high mobility pattern, with movements between Djara and the Nile Valley (approximately 150 km), is inferred [59].

In the small faunal assemblages from Hidden Valley Village site in the Farafra Oasis, dated to the 6^th^ millennium BC, sheep and goat bones are present, including a horn tip of a goat with twisted horns, and postcranial bones, clearly smaller than ibex or Barbary sheep [61]. Out of a total of 78 identified bones, it was possible to attribute about 15 to sheep or goat. No cattle were found, however Gautier (in press) suspects this may be due to the restricted size of the sample. For the Bashendi A phase in Dakhla Oasis (c. 6500–5600/5400 BC) cattle and goat are also reported, but without any detail on the type of remains and their numbers [62], [63], [64].

Lesur et al. [65] report on fauna from KS43 in the Kharga Oasis (4800–4400 BC). The presence of artesian wells made this area particularly favourable for occupation. The fauna is mainly composed of domesticates. Sheep/goat and cattle have been identified among them, but no pig. Remains of sheep/goat are the most numerous (about 1800 compared to less than 600 cattle bones) and sheep are more frequent than goats. Five dog bones were also identified. Game animals, ostrich (*Struthio camelus*), hare, Barbary sheep, but mainly gazelle are present as well. Fish remains are extremely rare and the few clariid bones found may represent the remains of imports from the Nile Valley [65]. This connection is confirmed by other evidence, including artefacts that indicate possible contacts with the Nile Valley (approximately 200 km distant) [66]. The few domestic plant remains recorded are pieces of cleaned grains, that are considered unlikely to be from local agriculture, but are probably also imports from the Nile Valley (Newton in [66]). Similarly, the large bivalves collected at the site are thought to originate from the Nile Valley [65]. Lesur et al. [65] tentatively interpreted KS43 as occupied during winter by mobile groups of pastoralists, based on the presence of a few foetal caprine bones that could be precisely aged from their length, combined with data on seasons of birth of recent caprines.

The most often cited early food production sites in the Western Desert are probably those from Nabta Playa and Bir Kiseiba. The sites cover a long sequence and have yielded the largest Neolithic faunal datasets in the Western Desert. Based on charcoal studied from one of the sites, an oasis-like vegetation around temporary bodies of water is reconstructed [67]. Nabta Playa and Bir Kiseiba are well known because of the oldest putative evidence for domestic cattle in Africa [55]. The earliest cattle remains may be as old as the late 9^th^-8^th^ millennium BC and recently, new finds of cattle from the same period were reported from Nabta Playa [68]. They were found at a new site with stratigraphic deposits, hearths and traces of dwellings. While these contexts seem well-dated through radiocarbon, there are no direct dates on cattle bones themselves, and more importantly a description of the bones, allowing an evaluation of their domesticated status, is not yet available. Only in the Middle (6100–5400 BC) and Late Neolithic (5400–4650 BC) are domestic sheep/goat added to the animal species spectrum of Nabta Playa and Bir Kiseiba [69]. Cattle are by then metrically distinct from aurochs and dogs are also present. Despite the presence of domesticates, throughout the Neolithic remains of hunted mammals, mainly hare and gazelles but also Barbary sheep dominate the faunal assemblages, although their importance decreases through time. Among the domestic animals, caprine are more numerous than cattle remains, and are mainly composed of sheep. No fish bones were found in the Nabta Playa-Bir Kiseiba area [69]. The archaeobotanical evidence points to the intensive use of a variety of wild plants [70]. No habitation structures were found for the earliest phases at Nabta Playa and Bir Kiseiba, but during the later Early Neolithic (Early Neolithic: ca. 8500–6100 BC), settlements became more stable, leaving behind remnants of wells, storages pits for plant food and houses [69]. Nevertheless, according to Wendorf and Schild [71], Neolithic occupation in the Nabta Playa-Bir Kiseiba area was never permanent.

Apart from the sites above, there are a few sites in the Western Desert of Egypt for which details are only available on the mammals [72]. These sites yielded wild animals only, except for site Glass Area 81/61 where three caprine bones were found (ca. 9000–5300 BC). In addition, there is Wadi Bakht (ca. 6800–4300 BC) in the Gilf Kebir where five cattle tooth fragments were recorded, along with about 20 gazelle and some unidentified bovid remains [73].

#### 2. Eastern Desert

Sodmein cave and the Tree Shelter are both located in the Eastern Desert about 40 km northeast of Quseir. There appear to have been several phases of Neolithic use at Sodmein, the first dated to about 6200–5800 BC, the second to 5400–5000 BC, and a third at around 4300 BC (radiocarbon dates can be found in [74], [75], [76]). This last period coincides with the radiocarbon dates from Tree Shelter at 4300 and 3700 BC. The area around the sites is today hyperarid but with occasional winter rains [77], [78]. During the Neolithic occupation, rainfall would have been more frequent and regular [78], resulting in a more lush environment [79]. Artefacts from Neolithic Sodmein and the Tree Shelter point to close parallels with the Middle and Late Neolithic from the Egyptian Western Desert [74], [75]. The faunal samples recovered from Neolithic layers at the sites are small. A few bones of marine fish have been found, as well as of hunted animals, including cat, rock dassie (*Procavia capensis*) and gazelle. Bones of domestic caprines are recorded for all phases, but their total number is not more than 13. Among these only goat could be identified to species level. No other domestic species are found. Apart from the scarce bone remains of domestic caprines, dung attributed to these animals was recovered [80]. In the most recent Neolithic phase at Sodmein this dung forms thick deposits. Both Sodmein and the Tree Shelter are presumed to have been used repeatedly, but for short periods only, as places where livestock was temporarily sheltered. Other Neolithic sites are not known from the Eastern Desert, which may be due to a lack of research, as this area has been much less intensively investigated than the Nile Valley and the Western Desert.

#### 3. Nile Valley

Sites are scarce in the Early and Middle Holocene in the whole of the Egyptian Nile Valley. Presumably the wet nature of the area made it unfavourable for human habitation [4]. Sites may also have been destroyed by recurrent Nile floods and buried underneath Nile alluvium, as some exceptional finds have shown [81]. Compared to the Nile Valley in Upper Egypt, which is like an elongated oasis, varying in width between more than 20 km in some places and only 1 km in others, the Delta of Lower Egypt is a lush and more humid area, nowadays stretching over a surface measuring 166 km long and 250 km wide [33].

At Merimde Beni Salama, in Lower Egypt, three phases of occupation are represented (see summary of archaeological data of Junker and Eiwanger in [26] and [82]). Few absolute dates are available but they indicate that the “Merimde Urschicht”, the earliest phase of occupation at Merimde, should probably be placed between ca. 4900 and 4700 BC, while the younger ones are from ca. 4600–4100 BC. The earliest occupation was a very light one only, but with evidence for postholes. For the later parts of the sequence, remains of oval houses are found. Cultivated crops at Merimde are emmer wheat (*Triticum turgidum* ssp. *dicoccon*), a free-threshing wheat (*Triticum aestivum/durum*) and hulled six-row barley (*Hordeum vulgare* ssp. *vulgare*) (see [83] for a detailed discussion). Based on records for storage facilities, it is suggested that farming became more important with time [26]. In the earliest levels at Merimde, sheep and goat predominate [12], followed by cattle and pig, which are about equally numerous. In higher levels the proportions between these domestic animals change. In level V, for example, pig becomes the main domestic animal. In general at Merimde, sheep outnumber goat (the proportion is about 20∶1). Large numbers of fish remains are also found. They represent 11.5% of the identified remains in level I and up to 45% in the later levels. Clariidae and tilapia are mainly represented by large, mature specimens. No details are available on the sampling techniques used and so small specimens may be missing due to a lack of sieving. Many remains of birds, which are typical winter visitors to Egypt, have been found at Merimde. Hunting played only a minor role. Species caught are hippo, hartebeest, gazelle and aurochs. The proportion of hunted animals at Merimde increases throughout the site's occupation. Hippo is the main game species and von den Driesch and Boessneck [12] have proposed that it was pursued to protect the fields from destruction by this large animal.

Saïs is contemporary with the later phases at Merimde [13], [84]. A Middle to Late Neolithic occupation (Saïs I), dated earlier than 4000 BC is attested, as well as a Late Neolithic one, dated around 4000 BC (Saïs II). In addition, there is also a Buto-Maadi Period phase at the site (ca. 3500 BC). The Saïs II deposits revealed features that may be the remnants of pits and post holes. During Saïs I, cattle and pig are about equally common, while domestic caprines are much less numerous [85]. During subsequent Saïs II, numbers of pig increase. Wild mammals and birds are rare. Fish are more common. In the Saïs I layers a fish midden was excavated. Its composition is largely dominated by clariid catfish, followed by tilapia [86]. Size reconstructions of these fish indicate on average smaller specimens than in Kom K and Kom W, but also show that small, young fish are present, as well as large, adult ones. Charred cereal remains were recovered, but were not identifiable [13]. The lithic studies by Phillipps [20] indicated higher mobility at Saïs than in the Fayum, and at Nabta Playa. Only a 10×10 meter area was excavated meaning that both the faunal and lithics assemblages come from a small area. This may explain the apparent contradiction between the high mobility indicated by the lithics and relatively large numbers of pig bones. We are more reluctant to draw parallels with historical examples in the Mediterranean of transhumant movements with pig herds [87].

Also corresponding in date with the later Neolithic occupation at Merimde is El Omari [88]. Its fauna [89] contains a lot of fish, mainly deep water species. Expressed in numbers of identified remains, domestic animals are the second most important. Pig, cattle and sheep/goat are present, in decreasing order of frequency. Hunted animals are mainly represented by hippo. As at Merimde, hippo hunting may have protected crops in the fields [89]. Evidence for settlement structures consists of postholes, as well as pits. Diverse refuse was found in these pits, which served presumably as storage pits before they came into disuse [90]. The archaeobotanical evidence shows the local cultivation of Near Eastern cereal and other crops, and include hulled barley (*Hordeum vulgare*) and a free threshing wheat that has been published by Helbaek [91]) as Club wheat (*Triticum aestivum* ssp. *compactum*), but may also be a landrace of Hard wheat (*Triticum turgidum* ssp. *durum*) with small-sized grain kernels [83].

In Upper Egypt, the Badarian site of Maghar Dendera 2 (4400–4250 BC) is the only one with faunal remains contemporary with the Neolithic sites in Lower Egypt. The site is interpreted as a temporary fishing camp, used at the end of the dry season, at the very beginning of the floods [92]. Fish are predominately deep water species, caught in the main river body. Apart from the large number of fish, remains of sheep, goat and some cattle were also found, but pig is absent. All later sites in Upper Egypt have ample pig [93] and the absence of this animal at Maghar Dendera may be related to the seasonal nature of the site. Maghar Dendera was probably used by a relatively small group of people who brought with them livestock animals, but not pig, to herd while they were away from their permanent base and homestead [92]. It is also clear that the composition of the faunal remains at this Badarian site is very different from that of later Predynastic sites in the Nile Valley [93]. Badarian people were probably mobile with a shift to more sedentary lifestyles in later phases. Faunal assemblages from the Nagada period (3800–2686 BC), following the Badarian, in Upper Egypt show a predominance of domestic animals, with fish being second in importance, but percentages are variable [93]. Some differences are apparent with contemporary sites in Lower Egypt, which have been related to different ecological conditions, such as the prevalence of goat over sheep and relatively lower numbers of pig, which are usually also smaller in size [93], [94]. At all Upper Egyptian settlement sites from the Predynastic, remains of hunted animals are not common and are mostly composed of gazelle [35].

## Discussion

### The introduction and propagation of domesticates over Egypt

**Figure 3:**
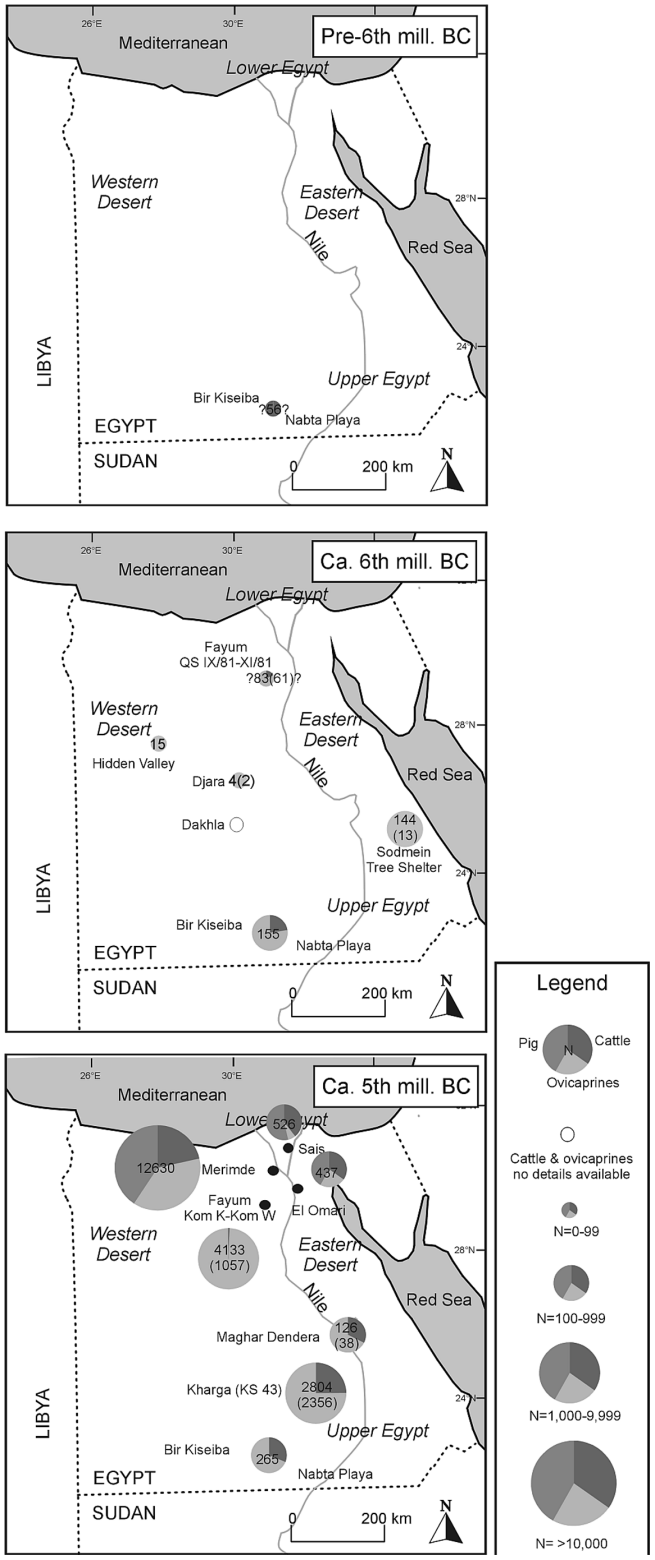
Overview of bone finds of domesticated animals in Egypt by broad chronological phase, based on data in Tables 4 and 6. Numbers between brackets exclude specimens from the imprecisely identified categories small and large bovids. Numbers with question marks are disputed or poorly contextualised.

The earliest possible domestic animals from Egypt, and Africa as a whole, are the putative domestic cattle from 8^th^ or even 9^th^ millennium BC deposits at Nabta Playa-Bir Kiseiba (Figure 3). However, both the dates of the finds and the domestic status of the cattle are still controversial [95]. While new finds of dwellings and hearths from this early period at Nabta Playa are well dated, the associated presence of domesticated cattle cannot be evaluated as the faunal data are not yet published [68]. The evidence from the Nabta Playa area remains isolated, with no contemporary remains recorded from neighboring areas. Claims for very early domesticated cattle in northern Sudan, starting from 7200 BC, which would have provided independent support for early finds in the Western Desert, were revised, as the bones come from large wild bovids instead of domesticated cattle [96]. If the 9^th^-8^th^ millennium BC date for domesticated cattle at Nabta Playa/Bir Kiseiba is correct, cattle keeping in Africa is as old as or older than in the Near Eastern domestication centres [2], [97]. Therefore, the putative domestic cattle from Nabta Playa-Bir Kiseiba are of crucial importance in the discussion on the existence of a local domestication of cattle in Africa (see for example [98]). However, based on a recent genetic study on over 1500 modern cattle individuals worldwide, it is hypothesized that extant African unhumped cattle are descendants of domesticated cattle from the Near East, but with a high level of admixture with local African aurochs [99]. This hypothesis of admixture remains speculative in the absence of genomes from African aurochsen.

Only from the Middle Neolithic onward (6100–5400 BC) do uncontroversial domestic cattle remains appear, now metrically distinct from aurochs, in the Nabta-Bir Kiseiba region. Their presence in larger numbers in combination with remains of domestic caprines leaves little room for doubt. Around 6000 BC domestic animals patchily appear in the archaeological record of different locations throughout the Western and Eastern Desert of Egypt (Figure 3). The Near Eastern origin of Northeast African caprines has been amply described before (e.g., [100]). The 6^th^ millennium BC dates, when secure domesticates appear, fit with the expansion of food producing economies from the Near East over the whole Mediterranean [2]. Numbers of bones are generally very small and, with the exception of Nabta-Bir Kiseiba and claims for cattle in Dakhla Oasis, only caprines are found. In the Western Desert sheep are mainly recorded and only goat is certainly present in the Eastern Desert. It has to be emphasized also that the 6^th^ millennium BC finds from Nabta Playa-Bir Kiseiba are exceptional, in terms of the presence of cattle and of the large numbers of domestic caprines compared to contemporaneous sites. The earliest domestic dogs have equally been identified from this area. For the 6^th^ millennium BC, there is no evidence for cultivated crops in Egypt. Instead, an intensive use of wild plants is indicated. Outside of the deserts, in the Fayum, domesticated animals are recorded from the second half of the 6^th^ millennium BC. There are not only indications for sheep\goat, but also cattle. However, the context of these early finds and their relation with the much more extensive 5th millennium BC archaeological remains is still unclear and nothing is known of the associated exploitation of plant food. As already indicated in the introduction, the 6^th^ millennium BC sites in the deserts of Egypt are the oldest on the African continent with undisputed evidence for food producing economies, in the form of mobile herding systems but without indications for agriculture. It should be emphasised, however, that the actual bone evidence for domesticates, upon which the inferences for these mobile herding systems is based, is scarce.

Only in the 5^th^ millennium BC do domestic animals appear in significant numbers outside the Nabta Playa-Bir Kiseiba area, as recorded at the Kharga and the Fayum Oasis and also at several sites in the Nile Valley. They now usually include domestic cattle, although at none of these locations are cattle numerically the most important domestic animals. From the 5^th^ millennium BC, dogs are also frequently found. If dogs indeed appeared as shepherd dogs together with small livestock from the Near East [100], [101], we may expect to find earlier evidence for dog. With the reclassification of Egyptian jackals as wolfs [21], [38], [39], a local domestication of dog is theoretically possible, although there is no concrete evidence that points in this direction. With the exception of the Western Desert sites, evidence for domestic pig as well as agriculture is present at all 5^th^ millennium BC sites in Egypt. Both were Near Eastern imports, meaning that renewed influences from the Near East probably have to be assumed.

### Two phases of early food production?

Although we must recognise the possibility that the patterns we are seeing are a consequence of archaeological visibility and choices in research strategies, the available evidence (Figure 3) suggests that there were at least two major phases of domestic animals and plants introductions from the Levant to northeastern Africa. During the first one, dated to the 6^th^ millennium BC and slightly earlier, sheep, goat and probably cattle were introduced. Bone evidence is very scarce outside the Nabta Playa-Bir Kiseiba area and, with the exception of the poorly understood 6^th^ millennium BC remains from the Fayum Oasis, it is only known from the deserts of Egypt. Game dominates the faunal assemblages. There is no evidence for plant cultivation and all faunal data point to mobile groups. It has been speculated, based on ethnographic studies, that for these early livestock keepers domestic animals may have served as a kind of “food reserve on the hoof” [102]. Their predictive availability would have been their main advantage over wild food resources. For this early phase, there are as yet no indications for the use of milk.

In the second phase, from the 5^th^ millennium BC onwards, domestic pigs and Near Eastern cultivated crops are attested. While the earlier introductions are typical for mobile herding systems, both pigs and agriculture are usually associated with less mobile subsistence styles. Starting in the 5^th^ millennium BC, remains of domesticated animals reach significant numbers in the faunal assemblages and sites with evidence for food production become ubiquitous. The expansion of evidence is mainly due to the appearance of sites in the Nile Valley. The large dung accumulations at Sodmein probably also reflect the expansion of the 5^th^ millennium BC. Neither locally grown crops nor pigs are known from the deserts, and with the exception of Kharga Oasis, remains of domesticates in the deserts remain low. There is no direct evidence for milk from the sites discussed but its extensive use has been proven at contemporary sites in Libya [58]. The changes in the 5^th^ millennium BC are probably to be correlated with climatic shifts: aridification after the Early and Mid Holocene climatic optimum [4] as well as changes in intensity of the Mediterranean winter rainfall [5].

### The variability of early food production in Egypt

Multiple factors bias the extent to which we can reconstruct former diet and economy from archaeozoological data. Many are taphonomic, including differential preservation and differential recovery depending on the animal species. However, the problems are also related to different animal sizes, one fish does not represent the same amount of food as one goat for example, and the ways in which animals were exploited, whether for meat or for milk, or a combination of these, in the case of caprines and cattle. The large caprine dung accumulations at Sodmein compared to the very low numbers of bones from the same layers, suggests that we should be cautious before translating poor numbers of bones into small herds. Also, even when quantitatively not important in the diet of the living human populations, animals may still be economically very important, as a food reserve on the hoof for example [102]. With this in mind, the key aspects in which the evidence for early food production inside modern Egypt shows variation will be discussed.

#### 1. Relative importance of domesticated animals

In the deserts, numbers of bones of domesticates are low throughout all sites and phases. The only exception is KS43 in Kharga Oasis (4800–4400 BC) where the archaeofaunal assemblage is predominantly composed of remains of domesticated animals. This is connected to the site's special local environmental conditions, with the presence of artesian springs in which wells could be dug. In the Fayum Oasis, bones of domesticates are generally not common relative total numbers of identified remains. In contrast, in the actual Nile Valley, domesticates represent large proportions of the total faunal samples, once they start to appear. After the Early and Mid-Holocene moist phase, the Nile Valley must have become a favourable area for stock keeping, with much easier access to food and fodder than in the deserts.

#### 2. Evidence for cattle

The Nabta Playa-Bir Kiseiba area stands out among the 6^th^ millennium BC evidence from the deserts, as the only one with domesticated cattle. Cattle are present at most Egyptian sites from the 5^th^ millennium BC but they are numerically usually not the most common domesticated species. In the Eastern Desert, cattle have not been evidenced at all. Cattle are known to be more difficult to keep than caprines, which are found more ubiquitously. They are an ecologically demanding species, tied to good pasture and sufficient drinking water [51].

#### 3. Evidence for pigs and cultivated crops

Evidence for pig and crops seems to concur in the Egyptian archaeological record. Where it appears, the presence of human groups with a less mobile lifestyle, presumably sedentary farmers, or at least contacts with such groups, can be supposed. Pigs and cultivated crops are first recorded at sites dated in the 5^th^ millennium BC in the Nile Valley and the Fayum Oasis. Apart from some grains obtained through contacts with the Nile Valley at KS43 in the Kharga Oasis, both pigs and crops are missing from the deserts. This is most probably due to unsuitable environmental conditions for local pig keeping and cultivation.

#### 4. Relative importance of wild game

Bones of wild game are mainly common at sites in the deserts, especially in the earliest phases. Nevertheless, only from 3500 BC do bones of hunted animals become less important in the Western Desert than those of domesticates [72]. A small component of wild game characterises faunal assemblages from the Nile Valley and the Fayum. Mainly the most easily available species appear to have been caught there. Both ethnographic and archaeological data from sub-Saharan Africa indicate that hunter-gathering is more important in the less suited areas for crop cultivation and/or stock keeping [103], [104], [105].

#### 5. Relative importance of fish

High numbers of fish are found at the sites in the Nile Valley. At two of these, Saïs and at Maghar Dendera 2, specialised fishing localities have been identified. More so than in the Nile Valley, in the Fayum the fauna is dominated by fish, which makes it very similar to the faunal assemblages from the earlier prehistoric phases in the area. Both the Nile Valley and the Fayum are characterised by the presence of a large water body and high numbers of fish are therefore not surprising. At the desert sites, fish are largely missing, apart from some specimens transported in from the Nile Valley.

#### 6. Mobility pattern

The general image that emerges for the mobility patterns is one of higher mobility in the deserts and lower mobility in the Nile Valley. It has been said that in the deserts, ecological conditions did not allow for sedentary lifestyles [59]. As part of their movements, members of the desert groups also seem to have made (occasional) visits to the Nile Valley, which represents a distance of up to 200 km. However, not many detailed studies into mobility patterns have been conducted for the Neolithic of Egypt. When these are undertaken results indicate that mobility is much more complicated than the generalised models predict [19], [20]. In the Fayum ongoing research is revealing that it is possible to discern complex regional mobility and settlement patterns wherein separate locations on the Fayum north shore may indicate how mobility varied among different places and times within the same region. The results also indicate the need to compare results from a number of proxies for human movement. Faunal analyses can help to indicate mobility with evidence for seasonally exploited resources. However, seasonal exploitation does not equal seasonal occupation. Pigs on the other hand, are usually associated with low mobility, but as this relation is not absolute, confirmation through other sources is necessary. The fauna thus illustrates the complexity of mobility patterns and the difficulty to reconstruct them from archaeological remains.

## Conclusions

An inevitable conclusion is that evidence for early stock keeping in Egypt is still very poor and that we are presumably facing a very biased sample due to uneven research intensity in different areas, differential preservation – with some deposits perhaps buried underneath Nile silt in the Nile Valley – and different mobility strategies. Even within one area, the Fayum Oasis, the data at present leave room for many questions. The discrepancy between the oldest date of 5400 BC for domesticated animals and the peak in evidence around 4500 BC is for example as yet unresolved and will be the subject of future research. Despite the poor amount of data, attempts at cultural-historical reconstructions of early food production in Egypt have been numerous. It is problematic that it is usually not clear that the reconstructions are based on such limited factual evidence.

The available data for the Egyptian Neolithic do not allow for fine diachronic reconstructions, and therefore the chronological subdivisions in this paper are necessarily very broad – typically on a millennial scale. Before the 6^th^ millennium BC, there is only the highly controversial evidence for domesticates. From the 6^th^ millennium BC, there is evidence for stock keeping, but it is clear that it is extremely patchy, with very few sites and usually not more than a handful of bones at each site. Food production in Egypt seems to first appear among mobile groups and the poor amounts of evidence may be related to this. Starting from the 5^th^ millennium BC, the amount of evidence increases dramatically and agricultural settlements appear. The data from the Fayum reflect this two-phase development, although for the second phase the evidence does not indicate agricultural “settlements”. For fine reconstructions of the rate of spread of domesticates, ideally (large sets) of direct dates on animal bones will be required. It remains to be seen whether this will be actually possible since radiocarbon dating of bone is difficult in Egypt due to the poor preservation of collagen.

The evidence from the Fayum and other areas of early food production in Egypt also shows that there is considerable regional variability. Much of the variation is probably due to the local environment and should be interpreted as local use of available resources. In the Fayum, the lake provided an important economic resource. However, environmental factors are insufficient to explain all variation. The increase of the role of food production was probably non-linear and complex, similar to the complexity in mobility strategies that is becoming apparent based on the study of different proxies for movement. The intricacy of early food production economies is increasingly shown for different parts of the world, with the island of Cyprus as a very clear example, where opportunistic shifts between hunting and herding are demonstrated [106]. As mentioned, the evidence for Egypt is insufficient for such detailed reconstructions as yet, which could demonstrate, for example, intermittent returns to an increased use of wild resources in certain periods. What is eminently clear, however, as shown through our work in the Fayum Oasis, is that in spite of the rapid increase in the destruction of these very vulnerable early sites, the evidence is still there, but requires very precise, painstaking and time consuming recording to provide the granularity necessary to improve the present state of knowledge.

## Supporting Information

Table S1 **Measurements (mm) on sheep/goat and cattle bones from Kom K and Kom W.**
(DOCX)

Table S2 **Size distributions (Standard Length, in cm) for clariid catfish and tilapia from Kom K and Kom W.**
(DOCX)

Table S3 **Fusion data of caprine bones from Kom K and Kom W.**
(DOCX)
