# Supplementary material for: New Archaeozoological Data from the Fayum “Neolithic” with a Critical Assessment of the Evidence for Early Stock Keeping in Egypt
Source: PLoS One. 2014 Oct 13;9(10):e108517. doi: 10.1371/journal.pone.0108517 (PMC4195595; doi:10.1371/journal.pone.0108517)
Supplement: Table S1 — Measurements (mm) on sheep/goat and cattle bones from Kom K and Kom W. (DOCX) [file pone.0108517.s001.docx]

**Table S1**. **Measurements (mm) on sheep/goat and cattle bones from Kom K and Kom W**

| Sheep/goat |  |  |  |  |  |  |  |  |  |
| --- | --- | --- | --- | --- | --- | --- | --- | --- | --- |
| **axis** | O-K-3-27 | K-7-17 |  |  |  |  |  |  |  |
| BFcr | 47.6 | - |  |  |  |  |  |  |  |
| SBV | 26.1 | - |  |  |  |  |  |  |  |
| LCDe | 58 | (65) |  |  |  |  |  |  |  |
|  |  |  |  |  |  |  |  |  |  |
| **scapula** | O-K-2-04 | W-1-19 | K-6-15 |  |  |  |  |  |  |
| GLP | 37,0 | 32.3 | - |  |  |  |  |  |  |
| LG | (30) | 26.4 | 26.4 |  |  |  |  |  |  |
| BG | 22.9 | 19.2 | 20.0 |  |  |  |  |  |  |
| SLC | 23.6 | - | 23.3 |  |  |  |  |  |  |
|  |  |  |  |  |  |  |  |  |  |
| **humerus** | K-1-02 |  |  |  |  |  |  |  |  |
| BT | (27) |  |  |  |  |  |  |  |  |
|  |  |  |  |  |  |  |  |  |  |
| **radius** | K-7-25 | W-4-28 |  |  |  |  |  |  |  |
| Bp | 33.1 | - |  |  |  |  |  |  |  |
| BFp | 30.2 | - |  |  |  |  |  |  |  |
| Bd | - | 25.9 |  |  |  |  |  |  |  |
|  |  |  |  |  |  |  |  |  |  |
| **ulna** | K-5-23 |  |  |  |  |  |  |  |  |
| LO | 38.6 |  |  |  |  |  |  |  |  |
| DPA | 24.1 |  |  |  |  |  |  |  |  |
| SDO | 21.1 |  |  |  |  |  |  |  |  |
|  |  |  |  |  |  |  |  |  |  |
| **pelvis** | W-2-02 | K-7-05 |  |  |  |  |  |  |  |
| LA | (30) | 28.3 |  |  |  |  |  |  |  |
|  |  |  |  |  |  |  |  |  |  |
| **femur** | K-7-39 |  |  |  |  |  |  |  |  |
| DC | 22.0 |  |  |  |  |  |  |  |  |
|  |  |  |  |  |  |  |  |  |  |
| **tibia** | K-07-13 | K-05-13 | K-03-27 |  |  |  |  |  |  |
| Bd | 28.8 | 25.5 | 25.2 |  |  |  |  |  |  |
|  |  |  |  |  |  |  |  |  |  |
| **patella** | W-2-06 |  |  |  |  |  |  |  |  |
| GL | 31.1 |  |  |  |  |  |  |  |  |
| GB | 23.6 |  |  |  |  |  |  |  |  |
|  |  |  |  |  |  |  |  |  |  |
| **os malleolare** | W-1-10 | W-2-18 |  |  |  |  |  |  |  |
| GD | 13.2 | 12,0 |  |  |  |  |  |  |  |
|  |  |  |  |  |  |  |  |  |  |
| **astragalus** | W-1-12 | O-K-6-36 | O-K-3-27 | C-K-6-22 | O-W-1-10 | K-6-31 |  |  |  |
| GLl | 31.4 | 30.2 | 30.2 | 27.3 | 27.2 | - |  |  |  |
| GLm | - | 29.1 | 28.2 | (28.2) | 26.4 | - |  |  |  |
| Dl | 18.3 | 17.2 | 17.2 | (15.5) | 15.8 | - |  |  |  |
| Bd | 21.2 | 20.0 | 19.5 | 19.0 | 18.2 | 18.2 |  |  |  |
|  |  |  |  |  |  |  |  |  |  |
| **os centrotarsale** | K-8-03 | O-K-3-32 |  |  |  |  |  |  |  |
| GB | 23.5 | 20.0 |  |  |  |  |  |  |  |
|  |  |  |  |  |  |  |  |  |  |
|  |  |  |  |  |  |  |  |  |  |
| **metarsus III+IV** | O-K-06-36 | O-K-06-36 |  |  |  |  |  |  |  |
| Bd | 26.2 | 26 |  |  |  |  |  |  |  |
|  |  |  |  |  |  |  |  |  |  |
| **metapodial** | O-W-1-10 |  |  |  |  |  |  |  |  |
| Bd | 26.7 |  |  |  |  |  |  |  |  |
|  |  |  |  |  |  |  |  |  |  |
| **phalanx 1** | C-K-6-9 | O- K-5-32 | O-K-6-36 | O-K-6-36 |  |  |  |  |  |
| GLpe | 42.4 | 37.8 | - | - |  |  |  |  |  |
| Bp | 12.9 | 10.9 | 12.1 | 11.6 |  |  |  |  |  |
| SD | 11.3 | 8.8 | - | - |  |  |  |  |  |
| Bd | 13,0 | 10.6 | - | - |  |  |  |  |  |
|  |  |  |  |  |  |  |  |  |  |
| **phalanx 2** | C-K-6-31 | O-W-6-11 | W-4-13 | O-K-3-27 | C-W-2-19 | O-K-6-36 | O-K-2-17 | O-K-6-36 | W-1-05 |
| GL | 25.6 | 25.5 | 25.4 | 23.9 | 23.3 | 23.0 | 22.6 | 22.2 | - |
| Bp | 15.1 | 12.1 | 11.1 | 11.7 | 12.2 | 11,0 | 11.1 | 12.4 | 11.5 |
| SD | 10.5 | 9.3 | 8.4 | 9.4 | 9.5 | 7.8 | 7.8 | 8.9 | - |
| Bd | 10.7 | 9.6 | 7.9 | 9.5 | 9.2 | - | 9.1 | 10.4 | - |
|  |  |  |  |  |  |  |  |  |  |
| **phalanx 3** | O-K-6-36 |  |  |  |  |  |  |  |  |
| DLS | 32.1 |  |  |  |  |  |  |  |  |
| Ld | 25.8 |  |  |  |  |  |  |  |  |
|  |  |  |  |  |  |  |  |  |  |
| Cattle |  |  |  |  |  |  |  |  |  |
| **phalanx 1 anterior** | K-7-41 |  |  |  |  |  |  |  |  |
| GLpe | 63.6 |  |  |  |  |  |  |  |  |
| Bp | 30.4 |  |  |  |  |  |  |  |  |
| SD | 25.7 |  |  |  |  |  |  |  |  |
| Bd | 29.7 |  |  |  |  |  |  |  |  |
|  |  |  |  |  |  |  |  |  |  |
| **metapodial** | K-3-06 |  |  |  |  |  |  |  |  |
| Bd | (55)* |  |  |  |  |  |  |  |  |
| Dd | (27)* |  |  |  |  |  |  |  |  |
| O: sheep; C: goat; K: Kom K; W: Kom W; numbers are trench number and unit number respectively | | | | | | | | |  |
| *: articulation not fused; **: articulation fusing; (): estimate | | | | |  |  |  |  |  |
| Measurements according to von den Driesch [52] | | | |  |  |  |  |  |  |
