# Supplementary material for: New Archaeozoological Data from the Fayum “Neolithic” with a Critical Assessment of the Evidence for Early Stock Keeping in Egypt
Source: PLoS One. 2014 Oct 13;9(10):e108517. doi: 10.1371/journal.pone.0108517 (PMC4195595; doi:10.1371/journal.pone.0108517)
Supplement: Table S2 — Size distributions (Standard Length, in cm) for clariid catfish and tilapia from Kom K and Kom W. (DOCX) [file pone.0108517.s002.docx]

**Table S2**. **Size distributions (Standard Length, in cm) for clariid catfish and tilapia from Kom K and Kom W**

|  | **Kom K** | **Kom W** |
| --- | --- | --- |
| Clariid catfish (Clariidae) | | |
| <10 | 1 | - |
| 10-20 | 5 | - |
| 20-30 | 16 | 8 |
| 30-40 | 36 | 12 |
| 40-50 | 81 | 23 |
| 50-60 | 81 | 28 |
| 60-70 | 59 | 20 |
| 70-80 | 50 | 14 |
| 80-90 | 11 | 5 |
| 90-100 | 7 | 3 |
| 100-120 | 1 | - |
| **Sum** | **348** | **113** |
| tilapia (Tilapiini) | |  |
| <10 | 1 | - |
| 10-20 | 120 | 21 |
| 20-30 | 1287 | 829 |
| 30-40 | 1399 | 832 |
| 40-50 | 145 | 71 |
| **Sum** | **2952** | **1753** |
| NISP by size category | | |
