# Supplementary material for: New Archaeozoological Data from the Fayum “Neolithic” with a Critical Assessment of the Evidence for Early Stock Keeping in Egypt
Source: PLoS One. 2014 Oct 13;9(10):e108517. doi: 10.1371/journal.pone.0108517 (PMC4195595; doi:10.1371/journal.pone.0108517)
Supplement: Table S3 — Fusion data of caprine bones from Kom K and Kom W. (DOCX) [file pone.0108517.s003.docx]

**Table S3. Fusion data of caprine bones from Kom K and Kom W**

| **Fusion age (months)** | **foet./** | **juv.** | **6-8** |  | **10** |  | **13-16** |  |  | **18-24** |  | **18-28** | | **30-36** | | **36** |  | **36-42** |  |
| --- | --- | --- | --- | --- | --- | --- | --- | --- | --- | --- | --- | --- | --- | --- | --- | --- | --- | --- | --- |
|  | **neon.** |  | scapula | | hum. dist. | | ph. 1 |  |  | os mc dist. | | os mp dist. | | fem. prox. | | rad. dist. | | hum. prox. | |
|  |  |  |  |  | rad. prox. | | ph. 2 |  |  | tibia dist. | |  |  | calc. |  | ulna prox. | | fem. dist. | |
|  |  |  |  |  |  |  |  |  |  |  |  |  |  |  |  |  |  | tibia prox. | |
|  |  |  | NF | F | NF | F | NF | fus | F | NF | F | NF | F | NF | F | NF | F | NF | F |
| Kom K - sheep or goat | 1 | 12 |  |  | 2 | 24 | 24 |  | 20 | 6 | 19 |  | 4 | 8 | 12 | 5 | 1 | 3 |  |
| Kom K - sheep |  |  |  | 1 |  |  | 2 |  | 5 |  |  | 1 | 2 |  |  |  |  |  |  |
| Kom K - goat |  |  |  |  |  |  |  |  | 2 |  |  |  |  |  |  |  |  |  |  |
| Kom W - sheep or goat | | 3 |  | 1 |  | 3 | 13 |  | 5 | 2 |  | 1 | 3 |  | 1 | 3 |  |  | 1 |
| Kom W - sheep |  |  |  |  |  |  |  | 1 |  |  |  |  | 1 |  |  |  |  |  |  |
| Kom W - goat |  |  |  |  |  |  |  |  | 1 |  |  |  |  |  |  |  |  |  |  |
| **Total** | **1** | **15** | **0** | **2** | **2** | **27** | **39** | **1** | **33** | **8** | **19** | **2** | **10** | **8** | **13** | **8** | **1** | **3** | **1** |
| NF: not fused, fus: fusing, F: fused | | | |  |  |  |  |  |  |  |  |  |  |  |  |  |  |  |  |
| Ages from Silver [52] |  |  |  |  |  |  |  |  |  |  |  |  |  |  |  |  |  |  |  |
